# Cambrian ‘sap-sucking’ molluscan radulae among small carbonaceous fossils (SCFs)

**DOI:** 10.1098/rspb.2023.0257

**Published:** 2023-03-29

**Authors:** Ben J. Slater

**Affiliations:** Department of Earth Sciences, Palaeobiology, Uppsala University, 752 36 Uppsala, Sweden

**Keywords:** Cambrian, molluscs, radula, herbivory, small carbonaceous fossils

## Abstract

Molluscs have produced an extensive fossil record, owing to the prevalence of robust biomineralized shells among this clade. By contrast, most other components of molluscan anatomy are seldom preserved. Importantly, little is known of the evolutionary history of the unique molluscan feeding apparatus—the radula. A scarcity of fossil radulae has hampered our understanding of the ancestral condition, and of the dietary ecology of early molluscs. The handful of known fossil radulae all point to early molluscs as simple deposit feeders that obtained food via rasping or scraping. This study reports microscopic radulae preserved as ‘small carbonaceous fossils' (SCFs) from Cambrian (Stage 4–Wuliuan, approximately 514–504.5 Ma) strata of Sweden. These rare fossil radulae offer novel insights into the feeding anatomy and ecology of early molluscs. Each radula comprises a uniseriate arc of (≤10) blade-shaped teeth, fringed by a slicing keel. This distinctive morphology is strikingly convergent with the radulae of extant sacoglossan heterobranch gastropods—such radulae are specially adapted for piercing the cell walls of green algal tissues to enable suctorial feeding on the cytoplasm contents. Discovery of analogous Cambrian radulae demonstrates this specialized form of herbivory had already evolved among molluscs more than half a billion years ago.

## 1. Introduction

Herbivory—the consumption of photosynthesizing autotrophs by animals—has been a profound force shaping the evolution of photosynthetic clades over the course of the Phanerozoic (538 Ma to present) [1–5]. Evidence of adaptations to the pressures of metazoan grazing are visible in the fossil record of Cambrian phytoplankton [6], in macroalgae since at least the Ordovician [7], and pervade the evolutionary history of land-dwelling tracheophytes [8].

Among grazers, molluscs have played a major role in exerting such herbivory pressures. Extant molluscs are important consumers of benthic macroalgae, and significant herbivores of terrestrial vascular plants [9,10]. In conjunction with the activities of sediment-churning bilaterians, early molluscan grazing may even have contributed to a shift in the nature of shallow marine benthic environments via the exclusion of widespread microbial mats and stromatolites [11–15].

Central to the profound ecological influence of molluscs are the specialized mouthparts comprising the molluscan radula. A uniquely molluscan feature, the radula is a multi-element feeding apparatus, which has been repeatedly adapted from its fundamental construction to fulfil a rich diversity of feeding functions, ranging from rasping, to predatory drilling, to filter-feeding [16].

Fossilized mouthparts can reveal detailed palaeobiological information about dietary ecology, and help to constrain when certain feeding behaviours first evolved [17–19]. Yet the fossil record of molluscan mouthparts—of radulae—is meagre. Molluscs have an extensive fossil record, yet it is one almost entirely consisting of the mineralized protective shells produced by many members of this clade [20]. By contrast, soft-bodied molluscs seldom fossilize, except in rare Lagerstätten deposits (e.g. [21]). Trace fossils provide some indirect evidence of molluscan ecology, including trails and shell borings [22]. Outside this limited window, however, the evolutionary history of molluscan diets remains largely inferred or unknown.

The few fossil examples of radulae that are known include rare instances of cephalopod radulae from the Ordovician [23] and Silurian [24], and the exceptionally preserved belt radula of an Ordovician stem aculiferan from the Fezouata Lagerstätten [25]. The oldest known unambiguous fossil radulae come from the Cambrian (Stage 4) Mahto Formation, Alberta, Canada [26]. These early Cambrian examples were recovered as acid-isolated microscopic organic remains, known as ‘small carbonaceous fossils’ (SCFs) [27].

Despite their microscopic size and fragmentary nature, SCFs can preserve delicate non-biomineralized animal structures outside rare Lagerstätten sites [27–31]. Such SCFs offer a relatively under-exploited source of fossil mouthparts from early in animal evolution. The emerging Cambrian SCF record has already yielded novel data on mouthparts and feeding apparatus that are otherwise invisible to the conventional fossil record [31]. For instance, exquisitely preserved mandibles captured among Cambrian SCF assemblages have extended the fossil record of particle-feeding crustaceans by approximately 100 Myr [30,32–35].

Here I describe isolated microscopic mouthparts among Cambrian SCFs that are identifiable as molluscan radulae. More specifically, these fossils represent uniseriate radulae, and exhibit a morphology characteristic of a specialized dietary ecology evolved for piercing-and-sucking the tissues of green algae. Their presence in Cambrian (Stage 4–Wuliuan) strata demonstrates that a relatively specialized form of herbivory and suctorial feeding—now found in derived sacoglossan heterobranch gastropods—was already present among Cambrian molluscs.

## 2. Material and methods

**Figure 1.**
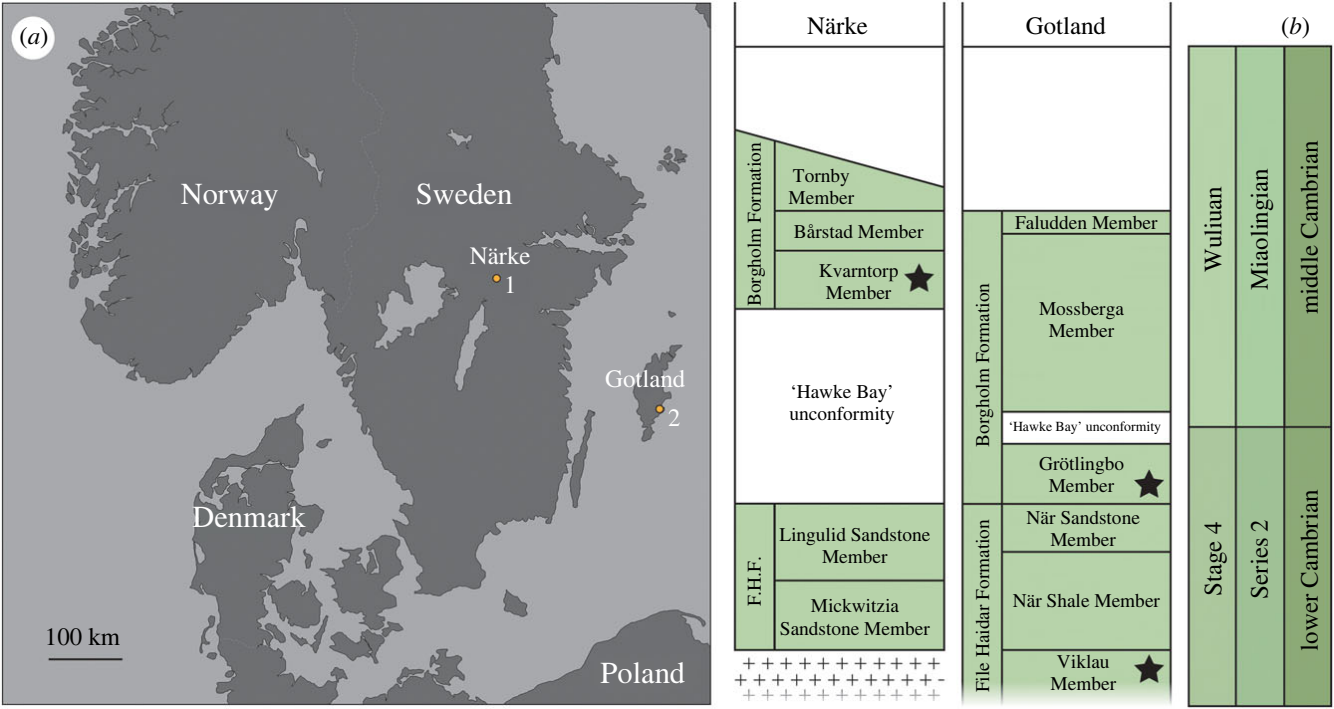
(*a*) Map of southern Scandinavia showing the position of sampled localities in Närke and Gotland (Sweden), (1) Kvarntorp locality, (2) När 1 core. (*b*) Distribution and stratigraphic subdivisions of Cambrian Stage 4–Wuliuan age Borgholm Formation and underlying Stage 4 File Haidar Formation across Närke and Gotland (after fig. 12 of [36]). Black stars denote members sampled for small carbonaceous fossils (SCFs) in this study. F.H.F. = File Haidar Formation.

The fossils were extracted from sampled outcrops of the Cambrian (Stage 4–Wuliuan) Borgholm Formation—a sequence of shallow-marine mudstones and sandstones developed over a broad area of southern Sweden (figure 1). Over most of its extent, the Borgholm Formation rests unconformably on siliciclastic rocks of the early Cambrian (Stage 4) File Haidar Formation, separated by a regional unconformity known as the ‘Hawke Bay event’ [36]. The Borgholm Formation is in turn overlain by the middle Cambrian to Tremadocian (Lower Ordovician) Alum Shale Formation. Based on subtle differences in lithology, the Borgholm Formation is partitioned into seven recognized subdivisions; the Grötlingbo Member, Mossberga Member, Faludden Member, Kvarntorp Member, Bårstad Member, Äleklinta Member and Tornby Member (figure 1). Several comparable specimens were also extracted from the Viklau Member (Cambrian Stage 4) of the underlying File Haidar Formation (figure 1) [36,37].

Mudstone samples (*n* = 7) of the Kvarntorp Member (Borgholm Formation) were collected from outcrop in Närke (at Kvarntorp Quarry, in spoil heaps adjacent to the water-skiing lake 59°7.1′ N, 15°17.3′ E). Kvarntorp Quarry material consists of green–grey glauconitic shales with bedding-plane visible specimens of the brachiopod *Acrothele granulate*, and was originally extracted from approximately 21 m from the base of the quarry section. Mudstones from the Grötlingbo Member (Borgholm Formation) were sampled from the När 1 core (Gotland) at 533.90, 537.52 and 547.92 m depth. Material from the Grötlingbo Member consists of fine-grained green–grey muds, with occasional *Teichichnus* burrows. A sample of green–grey bioturbated siltstone from the Viklau Member of the File Haidar Formation from 621.43 m depth in the När 1 core was also processed [37,38].

For each sample, approximately 50 g of rock was immersed in hydrofluoric acid (40% conc.) for 3–5 days before remaining organics were neutralized and rinsed over a 40 µm mesh sieve. Microfossils were hand picked from suspension in water via a low-manipulation extraction technique optimized for the recovery of delicate metazoan remains [27]. All samples produced abundant well-preserved organic microfossils, including a rich variety of acritarchs, filamentous microfossils and fragmentary metazoan remains. SCFs are permanently mounted on glass slides and stored in the palaeontological collections of the Museum of Evolution (PMU), Uppsala University, Sweden.

### (a) Fossil radulae

**Figure 2.**
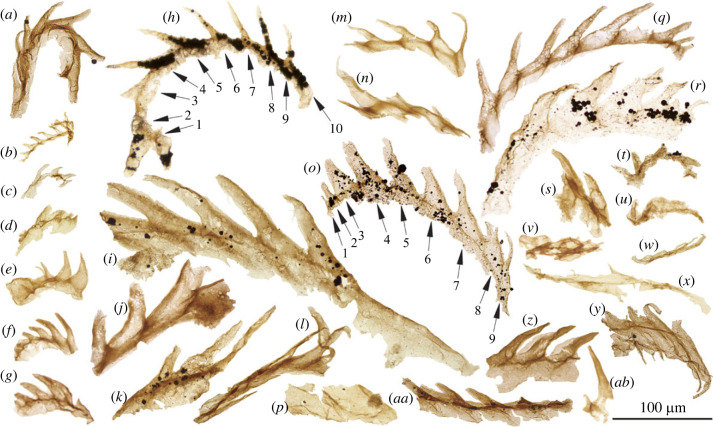
Acid-extracted Cambrian fossil radulae. The majority of specimens (*a–e, h–q, s–y, aa*) are from the Kvarntorp Member of the Borgholm Formation, Kvarntorp Quarry, Närke, Sweden (extracted material originally from approx. 21 m above base of section); (*g,r*) from Grötlingbo Member of the Borgholm Formation När 1 core, Gotland, Sweden (547.92 m depth); (*f,z,ab*) from Viklau Member of the File Haidar Formation När 1 core (621.43 m depth); (*a,h,o,q*) relatively intact portions of radular arc; (*v,w,x*) crushed/degraded forms; (*y*) possible ascus; (*ab*) possible tooth from a ‘boot-shaped’ radula. Slide numbers PMU 39412–39441. Numbered arrows in specimens (*h*) and (*o*) indicate numbers of individual teeth. Scale bar = 100 µm.

Among the thousands of recovered organic microfossils are a distinctive subset of metazoan-derived fragments (*n* = 41) that each comprises an isolated arc-shaped series (30–350 µm in length) of up to ten serially repeated blade-shaped tooth elements (figure 2). The tooth blades tend to increase in size in one direction (here interpreted as the anterior), and vary from sub-triangular to cone-shaped (individual teeth range from approximately 5 to 180 µm from base to tip). In some cases, the teeth are fringed by a thin flange or ‘keel’ that protrudes approximately 5–25% of the diameter of the tooth (figure 2*a,i*,*q*,*r*,*z*). The teeth articulate via interlocking knob-shaped extensions at the anterior and posterior of the tooth base (figure 2*j*). In some specimens, it is clear that each of the blade-like teeth possesses a narrow root and the teeth are connected by a thin basal membrane (figure 2*i*,*m*,*q*,*aa*).

Structures with a similar outline are found in the epipharynx of extant marine rotifers (compare figure 2*h* with figs 10, 13, 14 of [39]), the mandibles of copepod crustaceans (fig. 1K–O of [27]), and the gnathostomulid-like jaws of the Cambrian fossil *Amiskwia sagittiformis* (fig. 5 of [40]). In the case of rotifers and crustaceans, the underlying construction differs fundamentally from the SCFs described here, while in the latter case the paired tooth elements of *Amiskwia* are stouter and seemingly display a sclerotized tip that is absent among these SCFs.

**Figure 3.**
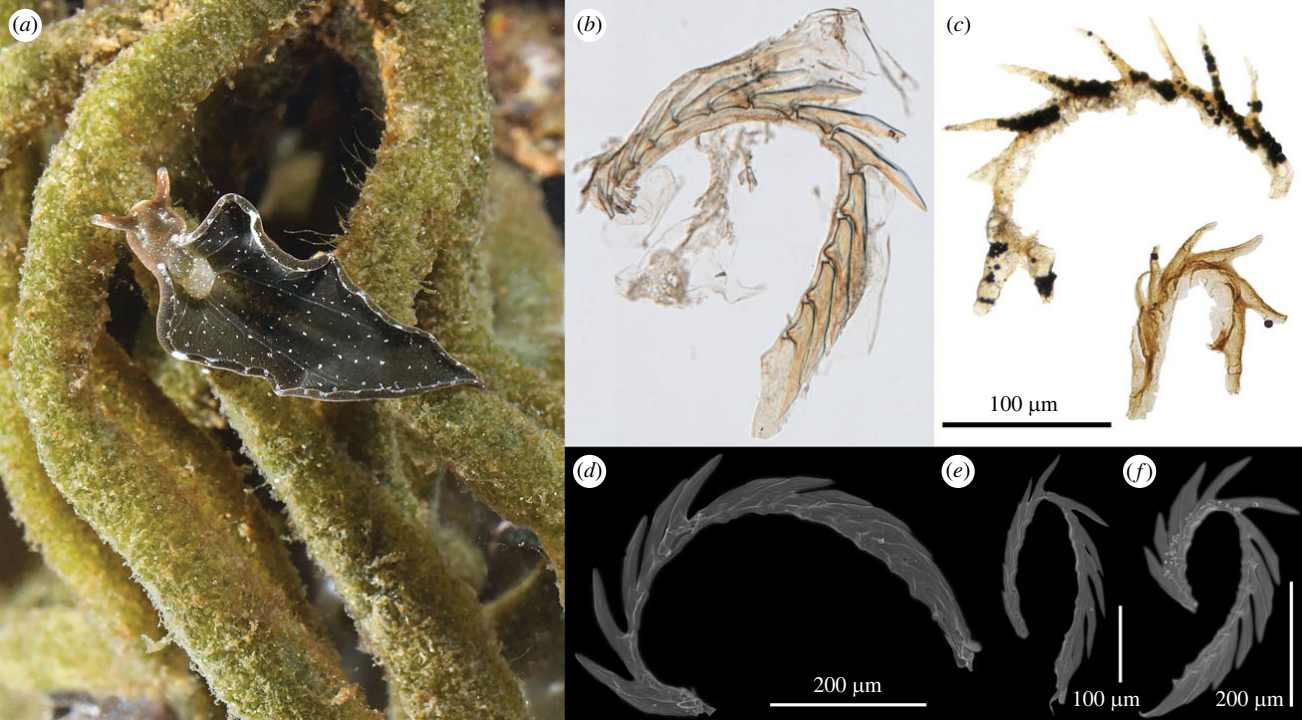
Comparison with the uniseriate radulae of extant Sacoglossa. (*a*) Extant sacoglossan *Elysia viridis* feeding on siphonaceous algae (courtesy of Jan de Vries and Sven Gould [60]); (*b*) radula from an *Elysia chlorotica* (Yale Peabody Museum of Natural History, photograph by D. J. Drew); (*c*) relatively complete portions of Cambrian fossil uniseriate radulae (this study); scanning electron microscope images of radulae from (*d*), *Elysia crispata*, (*e*) *Elysia papillosa*, (*f*) *Elysia canguzua* (*d*,*e*,*f* from [46], © Magnolia Press, reproduced with permission from the copyright holder).

A more precise correspondence is found among the uniseriate radulae of extant sacoglossan opisthobranchs (figure 3; [41–48]; fig. 2C–F of [49]). Among the Sacoglossa, blade-shaped teeth are arranged in an arc, and the teeth likewise possess a thinner, bladed keel on the dorsal or anterior (cusp) edge (see [43]; fig. 12 of [46]). In better preserved SCF specimens (e.g. figure 2*i*,*q*), a groove or ‘housing depression’ is apparent on the posterior margin of the tooth, which accommodated articulation with the neighbouring tooth in life—a feature also seen in the teeth of sacoglossan radulae. When viewed in this light, these SCFs conform to the morphology of uniseriate molluscan radulae, and are here interpreted as fragmentary portions of ascending and descending radular limbs.

An alternative possibility is that these SCFs represent isolated columns of teeth sourced from an originally distichous/polystichous radula—yet none exhibits any signs of compound arrangement, despite the large number of recovered specimens. Distichous and polystichous radulae that share some resemblance to these SCFs include the radulae of the patellid gastropod *Testudinalia testudinalis* (fig. 1*a* of [50]), as well as the radulae of certain limifossorid aplacophorans (e.g. *Scutopus variabilis* [51]) and Solenogastres (fig. 11*b*,*d* of [52], fig. 7*b* of [53]). Nevertheless, the teeth in these compound forms differ from the blade-shaped morphologies found in the SCFs (and in the radulae of sacoglossans), and instead comprise various spade-shaped or hook-shaped teeth adapted to scraping, often bearing prominent denticles (fig. 4 of [51]). Taphonomic flattening aside, the SCF tooth blades also appear to comprise a single type, and are oriented perpendicular to the base (figure 2) as seen in the tooth arcs of uniseriate radulae (figure 3).

Within each SCF tooth arc, the anterior and posterior teeth tend to be smaller. The ‘newer’, anterior teeth (descending limb) are less completely formed, and show no signs of wear (figure 2*q*), whereas the posterior teeth (ascending limb) are frayed at the tips (figure 2*o*), and in some cases are crumpled. These may represent worn older teeth still attached to the descending limb, for example, the tooth marked ‘10’ in figure 2*h*, and the tooth marked ‘9’ in figure 2*o*, as well as the crumpled teeth at the posterior of figure 2*a*,*r*. Here again there are similarities among the extant Sacoglossa, in which the worn-out teeth remain attached to the radula as an ‘ascus’ ([46], figs 5*c* and 26*c* of [47], fig. 4*c* of [54]). These retained teeth accumulate and are stored within a specialized pouch, the ascus sac. Compressed sections of blunted teeth among these Cambrian radulae (e.g. figure 2*y*) may represent isolated ascus-like portions.

Variations in the shape of the teeth among the recovered fossil radulae in part reflect taphonomic differences, but could also result from plastic adaptation to varied diets among a single molluscan taxon. Dietary-driven changes in radula morphology are well documented among extant molluscs [55], and particularly among the Sacoglossa (e.g. [56,57]). Nevertheless, the remainder of the organism's morphology is unknown, and therefore the possibility that these radulae are sourced from multiple taxa cannot be excluded. Macrofossils from the Wuliuan Borgholm Formation include a variety of molluscan shell remains (e.g. *Oelandia pauciplicata* [58,59]), among which could plausibly be the producer taxon of some of these radulae.

### (b) Functional morphology

**Figure 4.**
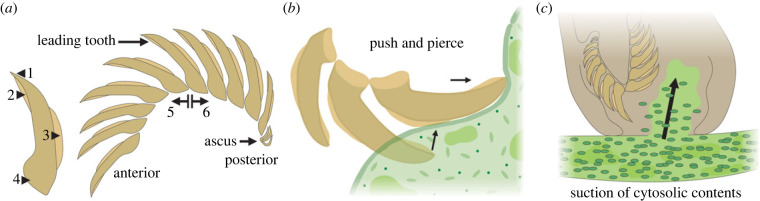
Schematic diagram of radula morphology and proposed functional morphology, including terminology used here. (*a*) Reconstructed articulated radula. Numbered arrows denote features of an individual tooth element including: (1) tooth tip; (2) bladed cutting edge on the cusp side of the tooth (extent variable among specimens); (3) dorsal keel, present in some specimens; (4) base; and (5) ascending and (6) descending limb. In life, teeth would have been secreted by odontoblast cells within a specialized radular sac, with new teeth formed at the anterior continuously moving the older teeth toward the posterior (where the oldest teeth may have been retained within an ascus sac). (*b*) Piercing and cutting action of the leading tooth, which produces a longitudinal slit in the algal cell wall (after fig. 4 of [43]). (*c*) Hypothesized suctorial feeding of cytosolic contents following breach of the algal cell wall, based on extant Sacoglossa (after various sources, including fig. 2A of [46]).

Radulae are adapted to particular dietary ecologies and exhibit morphology that reflects this [61]. Extant sacoglossan opisthobranchs possess a radular morphology specialized for a piercing-and-sucking habit, used in targeting algal tissues. Blade-shaped teeth are organized into a single row, and are adapted for cutting (rather than rasping), in order to pierce the algal cell wall and extract the cell sap (cytoplasm) and plastids via suctorial feeding [43,56] (figure 4). The vast majority of sacoglossans use this technique to feed on the multinucleate filaments of Siphonalea, a clade of macroscopic green algae (in certain extant Sacoglossa (*Elysia chlorotica*) the ingested plastids are even retained within a branching network of gut diverticula and used for photosynthesis, a process known as kleptoplasty [62,63]). Cambrian fossil radulae recovered here exhibit a similarly specialized morphology, with an arc of blade-shaped teeth clearly adapted for an equivalent piercing function.

The oldest known fossil Sacoglossa are bivalved shells from the Eocene [64]. Although most extant Sacoglossa lack shells (and those that possess shells are weakly biomineralized and live in erosive environments), a Cambrian record of Sacoglossa would nevertheless imply a vast ghost range (greater than 450 Myr). Further, the derived position of Sacoglossa would necessitate that more inclusive clades had already evolved by the middle Cambrian, implying substantial cryptic diversification of gastropods by this time. This, and the relative morphological simplicity of these Cambrian radulae (and those of extant sacoglossans), makes convergence a more likely explanation, rather than a deep origin of the Sacoglossa. Instead, these radulae reflect Cambrian molluscs that fed in an analogous manner by piercing and extracting the cell sap from filamentous green algae.

### (c) Mouthparts of *Wiwaxia*?

These fossil radulae from the Borgholm Formation are remarkably similar to SCFs recovered from younger mid-Cambrian (Drumian) sediments of Colombia that were originally compared to the transverse tooth bars of the iconic Cambrian taxon *Wiwaxia* (fig. 3 of [65]). The Colombian specimens were identified as candidate *Wiwaxia* mouthparts based on the recovery of co-occurring dorsal sclerites characteristic of *Wiwaxia* [65]. A link to *Wiwaxia* is somewhat undermined here by a lack of co-occurring sclerites in the Swedish material. Radulae are relatively common among preparations, yet the distinctive sclerites of *Wiwaxia* were not recovered. Wiwaxiid sclerites have been recovered from the File Haidar/Borgholm Formation boundary elsewhere in Sweden [66], but these do not co-occur with any known radula. If these mouthparts were sourced from wiwaxiids, then why concentrations of mouthparts would occur in the absence of the relatively robust dorsal sclerites (that would have been produced in much greater numbers) is puzzling. Biostratinomic sorting could explain such partitioning, but the radulae are recovered from parts of the Borgholm Formation separated by hundreds of kilometres (figure 1). Another explanation might be that these mouthparts are sourced from an *Odontogriphus*-like organism, which lacked a dorsal scleritome altogether.

Nevertheless, these isolated radulae differ from the mouthparts of articulated *Wiwaxia* (and the *Wiwaxia*-like mouthparts of *Odontogriphus* [67]) in some important respects. Though not bipartite, the mouthparts of *Wiwaxia* comprise a centrally positioned symmetrical tooth flanked by multiple tooth rows [68], quite unlike the single, uniseriate arrangement of teeth in the SCFs (figure 2*a*,*h*). The individual scoop-shaped tooth elements in the feeding apparatus of *Wiwaxia* and *Odontogriphus* also contrast with the blade-shaped teeth detected here. An assignment to *Wiwaxia*/*Odontogriphus* is therefore problematic. Pending the discovery of fully articulated material, it is practical then to simply resolve both the Colombian microfossils and Swedish specimens described here as portions of uniseriate radulae with a similar underlying construction. Whether such uniseriate radulae were borne by stem molluscs, or crown group molluscs during the Cambrian remains an unresolved question.

### (d) Cambrian radulae among organic microfossils

Radular fragments have occasionally been reported elsewhere among the emerging SCF record. Here these scattered reports are collated, including fragments figured among organic microfossils that were not originally identified as the remains of radulae.

SCFs described as radulae have been recovered from the Furongian Earlie (Pl. 1, figs 17, 18 and Pl. 2, figs 2–4 of [69]; fig. 2S of [70]) and Deadwood (fig. 2T,U of [70]) formations from Saskatchewan, Canada. A portion of a boot-shaped radula is known from the Miaolingian Bright Angel Shale of Arizona (fig. 14r of [71])—a more articulated segment of radula from this deposit has also been figured (fig. 2N of [27]). A possible radular fragment with similar boot-shaped scraping teeth has been recovered from the Wuliuan Kaili Formation of Guizhou, China (fig. 7M of [72]). Other SCF forms include the aforementioned ‘wiwaxiid radulae’ (interpreted here as uniseriate radulae) from mid-Cambrian (Drumian) sediments of Colombia (fig. 3 of [65])—a ‘denticulate metazoan SCF’ specimen from the Stage 4 File Haidar Formation of Sweden likely represents a similar type of radula to both these Colombian forms and those described here, but has a much broader basal membrane (fig. 10A of [66]). Fragments of potentially similar forms are also figured among organic microfossils from the Cambrian–Ordovician boundary of Dayangcha, China (pl. 88.14 of [73]), and from the Miaolingian Kistedalen Formation, Norway (fig. 24D of [74]).

**Figure 5.**
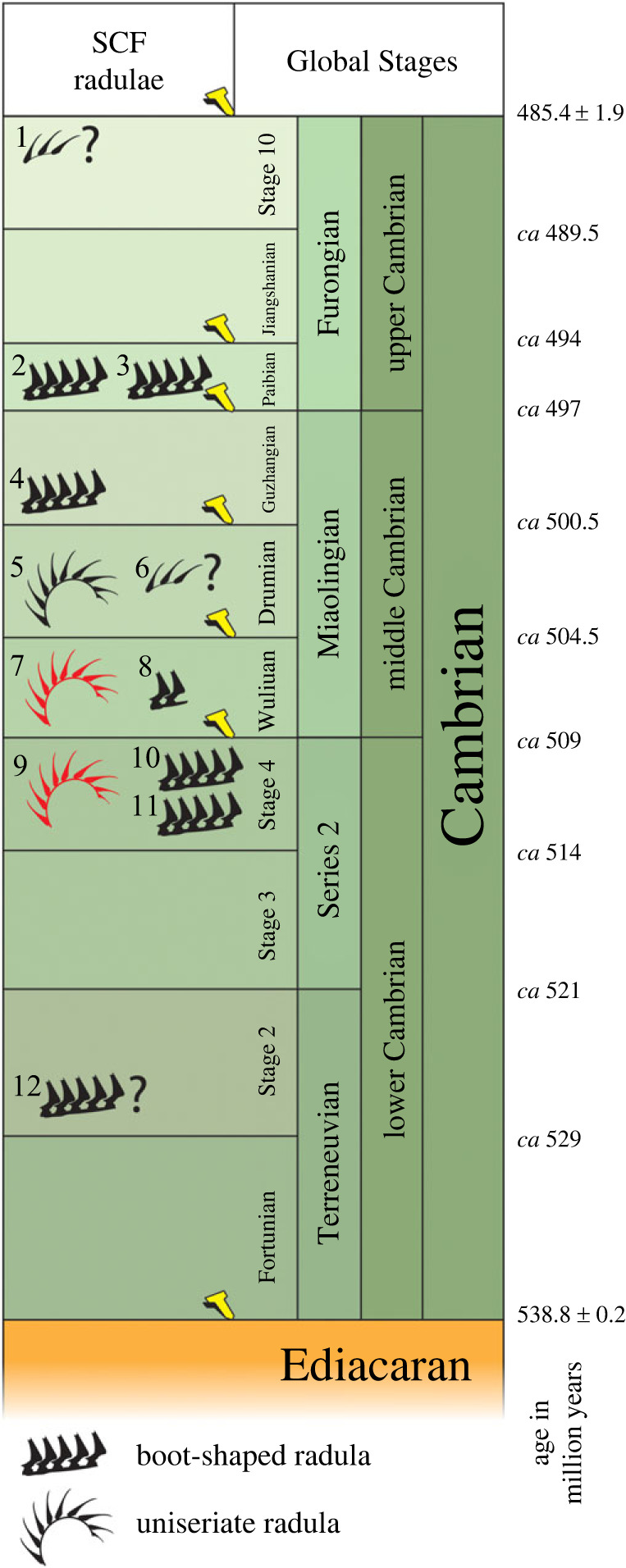
Stratigraphical occurrence of Cambrian molluscan radulae found as small carbonaceous fossils (SCFs). 1, Cambrian–Ordovician boundary, Dayangcha Beds, China [73]; 2, Earlie Formation, Furongian, Paibian, Saskatchewan, Canada [59,70]; 3, Deadwood Formation, Furongian, Paibian, Saskatchewan, Canada [59,70]; 4, Bright Angel Shale, Miaolingian, Arizona, USA [71,27]; 5, drillcore chippings, Miaolingian, Drumian of Colombia [65]; 6, Miaolingian Kistedalen Formation, Norway [74]; 7, Borgholm Formation, Stage 4–Wuliuan, Sweden; 8, Kaili Formation, Miaolingian, Wuliuan, Guizhou Province, China [73]; 9, File Haidar Formation, Stage 4, Sweden; 10, Mount Cap Formation, Series 2, Stage 4, Northwest Territories, Canada [27]; 11, Mahto Formation, Series 2, Stage 4, Alberta, Canada [26]; 12, Kessyusa Formation, Fortunian–Stage 2, Siberia, Russia [75].

Older fossil radulae include SCFs from the early Cambrian Stage 4 Mount Cap Formation (Colville Hills), Northwest Territories, Canada (fig. 2M of [27]), and the oldest described radulae in the fossil record are the specimens from the Cambrian (Stage 4) Mahto Formation, Alberta, Canada [26]. However, a semi-articulated portion of what appears to be a radula has been recovered among SCFs from the Fortunian–Stage 2 Kessyusa Formation of Siberia (fig. 25K of [75]). This latter example bears a striking resemblance to the boot-shaped SCF radulae reported from younger strata and would push back the record of fossil radulae even further, to the earliest Cambrian Terreneuvian Series (figure 5).

With the exception of the specimens from Colombia, these previously detected SCF radulae display a different construction from those described here, and were likely adapted for scraping and rasping (though bundled morphologies from the Mahto Formation could have been used in filtering/straining). Despite the patchiness of the current record (figure 5), it is evident from the presence of various uniseriate, distichous and polystichous forms that a degree of radular diversification occurred during the Cambrian.

### (e) Cambrian herbivory

Despite its importance, the origins of herbivory remain poorly understood. Records of Cambrian early metazoan ecosystems are filled with accounts of filter/suspension-feeding primary consumers, scavengers and carnivores, but display comparatively little documentation of grazers or active herbivores [76–78]. Indeed the slicing and grasping jaws and feeding structures of predators account for the majority of known Cambrian mouthparts [79]. This dearth of herbivores appears characteristic of the early Palaeozoic, and has led to the hypothesis that macrophagous herbivory targeting multicellular plant tissues (as opposed to microphagous consumption of protists and cyanobacteria) was a belated innovation [77]. Nonetheless, direct herbivory is frequently difficult to infer from mineralized hard parts, and traces of herbivory are poorly documented and/or hard to recognize in fossilized macroalgae.

Fossil macroalgae are known from sedimentary rocks dating as far back as the Tonian Period [80,81]. Despite their overlap with the animal fossil record in the Ediacaran and Cambrian, such macroalgae show little sign of counteradaptation to herbivore grazing until the Ordovician [7]. Accumulated records of fossilized terrestrial vegetation show similar lag-times; although fossils show that some plant tissues were targeted early in trachaeophyte evolution (e.g. sporangia and spores), certain tissues and organs appear to remain unexploited by herbivores until tens of millions of years after their origin [8]. In the case of macroalgae, this delay may be accounted for in part by an initial dependence on cyanobacteria, and/or targeting of algal cell cytoplasm, perhaps followed by an expansion of direct grazing and maceration of algal tissues during the Ordovician.

Herbivory comprises more than simply the maceration and consumption of plant material and also encompasses a broad swath of other feeding modes that exploit plant tissues for nutriment. Early in animal evolution, the dominant forms of herbivory may have been microphagous consumption of cyanobacteria [77], but the recovery of specialized radulae among Cambrian SCFs suggests that piercing-and-sucking type feeding on green algae may be an overlooked component of primary consumption in early metazoan ecosystems.

## Data Availability

This article has no additional data.
